# Supplementary material for: Chromothripsis during telomere crisis is independent of NHEJ, and consistent with a replicative origin
Source: Genome Res. 2019 May;29(5):737–49. doi: 10.1101/gr.240705.118 (PMC6499312; doi:10.1101/gr.240705.118)
Supplement: Supplemental Material [file supp_gr.240705.118_Supplemental_file_1.zip › contigs/annotated_contigs/DB92/contig.4.DB92_length_665_mean_cov_5.23308270677.docx]

**DB92_length_665_mean_cov_5.23308270677**

```
AAGATTGGGGTAAGTTATTTAATCCTTTTCTCAAAAACCTAAGGGTTTTTTTGGGGATATGCTCAGGTGGCACAGCAAGCCACAAGATA
 >chr13:80931415-80931560 + E=2e-72 p=0e+00
ACATGGGCTATCTAACATTATTTCAATTTACATATATATATATATATATATATATA|TATATATATATATAT|AAATAATTTTTCAGGC
                                                                        >chr13:80932052
AATGAGACTCTGGCTCTTTCCATGTTTAGTCTATTAGGATTTGTGTTCTCAGGGAGCACTTAGGGCTACTGAGGCCTTGAGTCAATGTT
-80932304 - E=7e-140 p=0e+00
CAGACCCTGGGTTGGGTGCTTACTAATCGCTAAACTCCTAAGCCATAAGCTGACCCAACAAACCTTCCTAAAAACAGAGGAGCAGCCGG

GCT|CAGTGGCTCACGCCTGTAATCCCAGCACTTTGGGAGGCCGAGGCAGGCGGATCAC|CTGAGGTCGGGAGTTCAGGACCAGCCTGA
    >chr11:77335591-77335817 + E=4e-85
CCAACATGGAGACACCCAATCTCTACTAAAAATACAAAATTAGCCAGGCAGGGTGGCACATGCCTGTAATCCCAGCTACTTGGGAGGCT

GAGGCAGGAAAATCGCTTGAACCTGGGAGGCGGAGGTTGCAGTGAGCAGAGAC|TGTCCCATTGC|ACTCTAGCCTGGGTGACAAGAGC
                                                                 >chr11:118292468-11829
GAAACTCCGTCTCAAAAAAAAAGTGTCCATTAATTTTGGCAAATAGCC2541 + E=4e-26
```
